# Deep radiomics-based survival prediction in patients with chronic obstructive pulmonary disease

**DOI:** 10.1038/s41598-021-94535-4

**Published:** 2021-07-26

**Authors:** Jihye Yun, Young Hoon Cho, Sang Min Lee, Jeongeun Hwang, Jae Seung Lee, Yeon-Mok Oh, Sang-Do Lee, Li-Cher Loh, Choo-Khoon Ong, Joon Beom Seo, Namkug Kim

**Affiliations:** 1 Department of Radiology, Asan Medical Center, University of Ulsan College of Medicine, Seoul, South Korea; 2 Department of Radiology, Korea University Guro Hospital, Korea University College of Medicine, Seoul, South Korea; 3 Department of Medicine, University of Ulsan College of Medicine, Seoul, South Korea; 4 Department of Pulmonary and Critical Care Medicine and Clinical Research Center for Chronic Obstructive Airway Diseases, Asan Medical Center, University of Ulsan College of Medicine, Seoul, South Korea; 5 Department of Medicine, RCSI & UCD Malaysia Campus, Penang, Malaysia; 6 Department of Convergence Medicine, Asan Medical Institute of Convergence Science and Technology, Asan Medical Center, University of Ulsan College of Medicine, Seoul, South Korea

**Keywords:** Biomedical engineering, Prognosis

## Abstract

Heterogeneous clinical manifestations and progression of chronic obstructive pulmonary disease (COPD) affect patient health risk assessment, stratification, and management. Pulmonary function tests are used to diagnose and classify the severity of COPD, but they cannot fully represent the type or range of pathophysiologic abnormalities of the disease. To evaluate whether deep radiomics from chest computed tomography (CT) images can predict mortality in patients with COPD, we designed a convolutional neural network (CNN) model for extracting representative features from CT images and then performed random survival forest to predict survival in COPD patients. We trained CNN-based binary classifier based on six-minute walk distance results (> 440 m or not) and extracted high-throughput image features (i.e., deep radiomics) directly from the last fully connected layer of it. The various sizes of fully connected layers and combinations of deep features were experimented using a discovery cohort with 344 patients from the Korean Obstructive Lung Disease cohort and an external validation cohort with 102 patients from Penang General Hospital in Malaysia. In the integrative analysis of discovery and external validation cohorts, with combining 256 deep features from the coronal slice of the vertebral body and two sagittal slices of the left/right lung, deep radiomics for survival prediction achieved concordance indices of 0.8008 (95% CI, 0.7642–0.8373) and 0.7156 (95% CI, 0.7024–0.7288), respectively. Deep radiomics from CT images could be used to predict mortality in COPD patients.

## Introduction

Chronic obstructive pulmonary disease (COPD) is a chronic inflammatory lung disease that causes airflow limitation and symptoms include shortness of breath, frequent coughing or wheezing, and excess mucus (sputum) production. COPD is a major cause of chronic morbidity and mortality throughout the world; many people suffer from this disease for years and die prematurely from it or its complications^1^. Pulmonary function tests (PFTs) are currently used to diagnose and classify the severity of COPD, but they cannot fully represent the type and range of pathophysiological abnormalities of the disease. In particular, PFTs tend to be relatively insensitive to early COPD symptoms and subtle symptom changes^2^. Furthermore, patients with similar PFT values may exhibit completely different clinical and radiologic phenotypes.

Medical imaging, which provides multiparametric morphologic and functional information, plays an increasingly significant role in precision medicine. Chest computed tomography (CT) provides in vivo visual information that can be used to investigate structural and underlying pathophysiologic changes in COPD patients, and thus allows analysis of primary features of COPD including morphologic characteristics and the distribution of both emphysema and small airway disease^3–6^. However, qualitative CT assessment by radiologists, which has been the mainstream method for acquiring information from CT scans, is prone to inter-reader, and sometimes even intra-reader, variability, limiting its application to broad clinical and experimental settings^7^. Therefore, the need for more objective CT-based measures has grown significantly.

Radiomics has been proposed to explore the correlation among medical images, other -omics, and clinical parameters, and interest in its application has been growing since it has the potential to provide significant interpretive and predictive information to support decision making. Radiomics is the process of extracting high-throughput quantitative features from radiographic images and building predictive models relating image features to genomic patterns and clinical outcomes^8^. In the past few years, a number of radiomics models have been proposed for tumor classification^9–11^, survival prediction^12,13^, and recurrence prediction^14,15^.

In radiomics-based analysis, high-throughput feature extraction (i.e., radiomics) is a critical task. In previous studies, most extracted features were designed by hand or explicitly. In the field of COPD, quantitative CT imaging methods have been proposed to provide more precise and reproducible estimates of the severity and distribution of emphysema and airway disease^16^. In the early days of quantitative imaging biomarkers, research was weighted toward quantification of emphysema, and there has recently been an increasing number of publications targeting the airway component of COPD, which includes direct airway parameter measurements and quantification of air trapping as functional manifestations of small airway disease^17^. Moreover, quantitative pulmonary vascular features turned out to be associated with COPD severity and emphysema extent^18^. Although the number of handcrafted features can reach tens of thousands, these features are shallow and low order. They may not fully characterize image heterogeneity and may limit the potential of radiomics models. Therefore, it is necessary to assess deeper and higher-order features that may improve the predictive performance of radiomics models.

Recently, the performance of deep learning has been intensively demonstrated in computer vision. In particular, a convolutional neural network (CNN), which uses a trainable filter bank with an extensive weight-sharing scheme, can quickly outperform state-of-the-art approaches in many computer vision tasks, including image classification and segmentation^19–22^. These deep learning-based approaches have also impressive results for CT analysis in COPD^23^.

CNN can be incorporated into current radiomics models by extracting many deep features from hidden layers^24,25^. These deep features, extracted not by feature engineering (handcrafting) but by feature learning, could contain more representative and high-level medical image information and provide more predictive patterns compared to handcrafted features. In this paper, we propose a deep feature-based radiomics model for predicting the overall survival of COPD patients.

## Materials and methods

### Patients

There were two groups of patients: (1) a discovery cohort with 344 patients from the Korean Obstructive Lung Disease (KOLD) cohort and (2) an external validation cohort with 102 patients from Penang General Hospital in Malaysia. The inclusion and exclusion criteria for the discovery cohort have been published previously^26^. In short, patients over 18 years of age with chronic respiratory symptoms and airflow limitations or bronchial hyperresponsiveness were included. Between June 2005 and April 2012, 344 patients with an established COPD diagnosis and available volumetric chest CT scans taken at the time of registration were enrolled. Subjects underwent PFTs within two weeks of volumetric chest CT image acquisition. Baseline clinical characteristics, PFT results, six-minute walk distance (6MWD) results, and survival information were documented for all patients. Our institutional review board approved this study, and written informed consent was obtained from all patients. The external validation cohort included patients in the chest clinic of the 1200-bed Penang General Hospital, which is part of the Asian Network of Obstructive Lung Disease^27^. All patients with stable COPD who were referred to or followed up were invited to participate. Prospective data of 112 eligible COPD subjects was available for mortality analysis. Inclusion and exclusion criteria have been published previously^28^. Of 112 patients initially eligible, 10 were excluded due to poor CT image quality. The median follow-up time was 1000 days (range, 60–1400). Quantitative CT and clinical demographic data were collected at the time of study entry. Written informed consent was obtained from all participants. Research and ethical approval was obtained from the National Research and Ethics Committee of Malaysia (NNMR-13-313-15138). All methods were performed in accordance with the relevant guidelines and regulations.

### Volumetric chest data acquisition

Discovery cohort volumetric chest CT scans were obtained using 16- or 64-slice multidetector CT (MDCT) scanners produced by two different manufactures (259 CT scans using SOMATOM Sensation 16 or SOMATOM Definition AS from Siemens Healthineers AG, Bonn, Germany; 85 CT scans using Philips Brilliance 16, 40, or 64 from Philips Medical Systems, Best, Netherlands). Patients were scanned craniocaudally in the supine position during full inspiration. Routine administration of intravenous contrast media was not required for image acquisition using either type of scanner. CT scan parameters were: collimation of 16 × 0.75 mm, effective mAs of 100, kVp of 140, and pitch of 1. CT data were reconstructed at a 0.75-mm slice thickness and 0.7-mm increment using a B30f kernel for Siemens scanners and a 0.8-mm slice thickness and 0.8-mm increment using a standard reconstruction algorithm for Philips scanners.

External validation cohort volumetric chest CT scans were obtained using a 64-slice MDCT scanner (SOMATOM Sensation 64; Siemens Healthineers AG, Forchheim, Germany) at the Loh Guan Lye Specialist Centre in Penang, Malaysia. CT scans were obtained using standardized protocol from the Research Institute of Radiology of the Asan Medical Center in Seoul, South Korea^4,29^. The CT scan parameters were a collimation of 0.75 mm, effective mAs of 100, kVp of 140, and pitch of 1. Patients were scanned craniocaudally in the supine position during full inspiration. Images were reconstructed using a soft kernel (B30f; Siemens Healthineers AG) from thoracic inlet to lung base. Image quality and protocol compliance were verified by the Asan Medical Center.

### Deep features extraction

**Figure 1:**
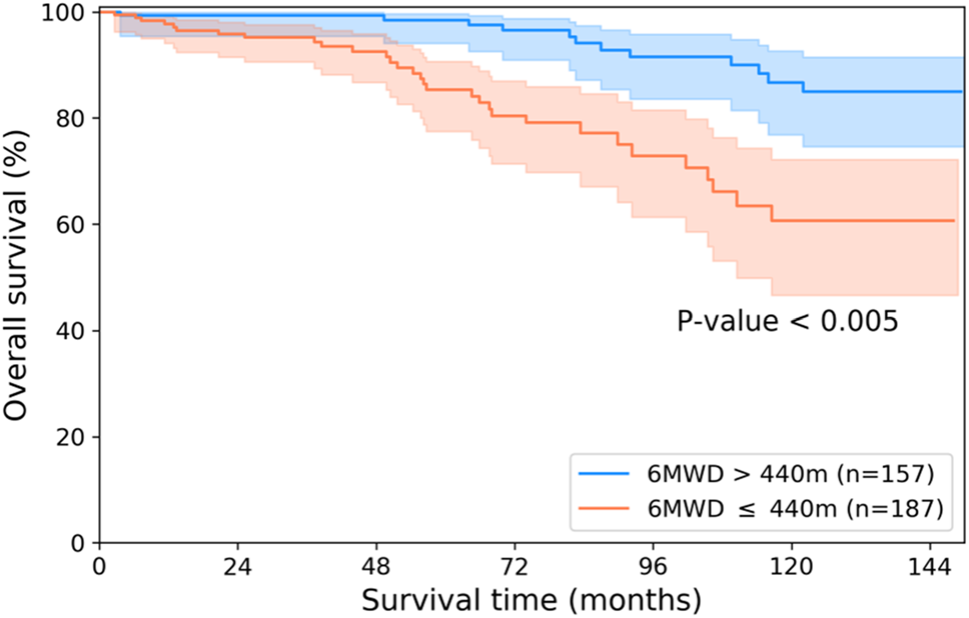
Kaplan–Meier survival curves for patients with 6MWD over and less than 440 m in discovery cohorts. We designed and trained a CNN-based binary classifier to obtain high-level representative information from chest CT that can predict mortality in patients with COPD. This CNN-based binary classifier was based on the 6MWD testing results, and the discovery cohort was divided into two groups with the value of 440 m: 157 low-risk patients and high-risk 187 patients. The estimated cumulative survivals of these two groups were significantly different.

**Figure 2:**
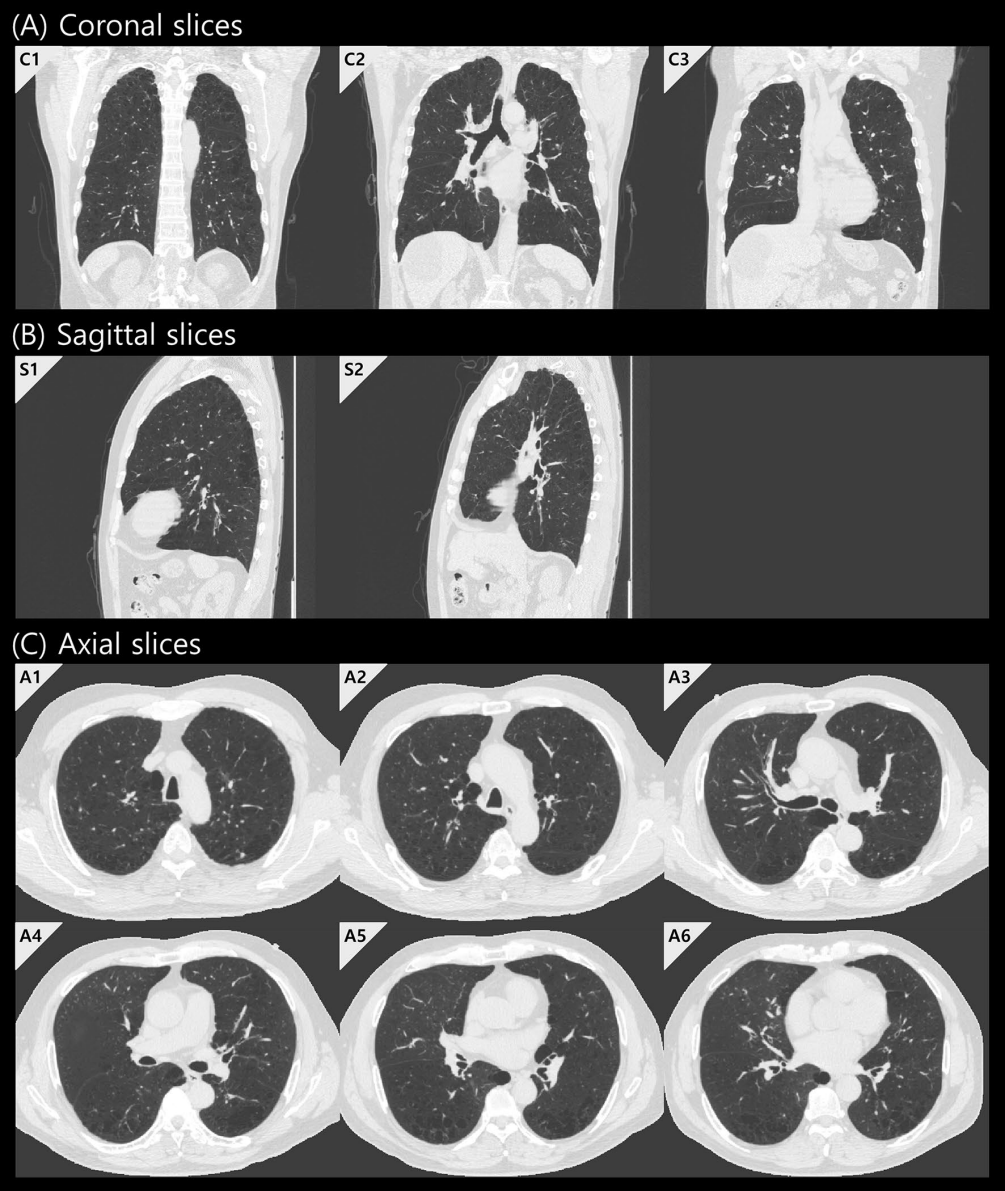
Convolutional neural network (CNN) model input to extract deep features. To extract deep features, 11 computed tomography slices were selected for CNN model input based on predetermined anatomic landmarks. (**A**) Coronal slices at the vertebral body (C1), center of the tracheal carina (C2), and superior vena cava (C3). (**B**) Sagittal slices at the center of the left (S1) and right (S2) lung. (**C**) Axial slices at two upper slices (A1, A2), center slice (A3), and three lower slices (A4, A5, A6) at 2-cm intervals at the center of the tracheal carina.

**Figure 3:**
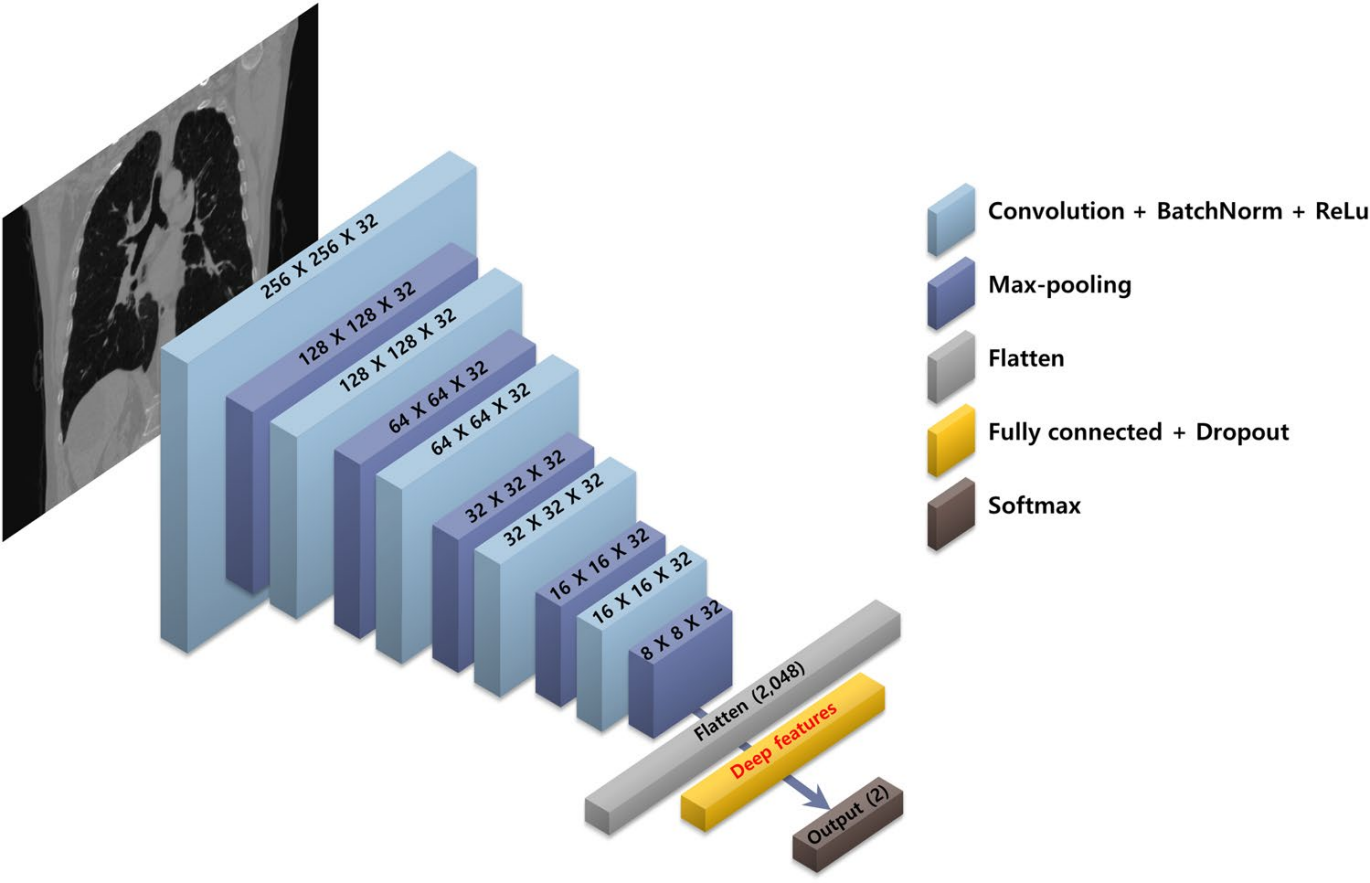
Convolutional neural network (CNN) model architecture. CNN architecture for classification of six-minute walk distance test results (> 440 m or not) to extract deep features. Each of the 11 selected slices was trained separately using the same architecture, which was designed to have five convolution blocks and one fully connected layer. Deep features were obtained by normalizing the information of the last fully connected layer (yellow). Each convolution block consisted of a convolutional layer followed by batch normalization (*BatchNorm*), rectified linear unit activation (*Relu*), and max-pooling.

We trained a CNN model to obtain high-level representative information from medical images, and high-throughput image features (i.e., deep radiomics) were directly extracted from the last fully connected layer. The CNN performed binary classification based on 6MWD testing, one of the most important factors for evaluating the ability to perform activities of daily living. According to the 2015 European Society of Cardiology/European Respiratory Society Guidelines for diagnosing and treating pulmonary hypertension, a 6MWD result > 440 m is one of several factors associated with low one-year mortality^30^, so 440 m was selected as the optimal threshold. Therefore, training the classifier with deep learning using 6MWD results > 440 m could not only reveal known prognostic features but also potentially identify previously unknown ones. With the value of 440 m, the discovery cohort consisted of 157 patients with 6MWD over 440 m and 187 patients with 6MWD less than 440 m, not causing data imbalance in binary classification. The baseline clinical characteristics and the Kaplan–Meier-estimated cumulative survivals of these two groups were compared in Table 1 and Fig. 1, respectively, and a CNN-based binary classifier was designed to distinguish these two groups. Due to the nature of the discovery cohort and the limited processing capabilities of existing graphical processing units, the whole slices were not available. Even if it is possible to utilize the whole slices with reduced resolution, there should be a sufficient number of datasets to be able to train them. Considering the number and mortality of our dataset, training the whole slices was difficult. So, with the advices of expert radiologists, 11 representative CT slices were selected for each patient based on predetermined anatomic landmarks and used for CNN input: (1) three coronal slices at the vertebral body, center of the tracheal carina, and superior vena cava, (2) two sagittal slices at the center of the left and right lung, and (3) six axial slices (two uppers, center and three lowers) at 2-cm intervals at the center of the tracheal carina (Fig. 2). Anatomic landmarks were selected via consensus of two thoracic radiologists (each with more than 20 years of experience) with the idea that information on the distribution of emphysema or airway changes should be necessary, and 11 CT slices in both cohorts were manually selected by an experienced research assistant, and then finally confirmed by a thoracic radiologist. In the case of axial slices, 2-cm intervals were adopted as a way of obtaining the maximum information with a small number of slices. To classify low-risk patients based on 6MWD results, the CNN was designed to have five convolution blocks and one fully connected layer (Fig. 3). Each convolution block consisted of a convolutional layer with 32 learnable filters followed by batch normalization, rectified linear unit activation, and max-pooling. To prevent overfitting, a connection dropout probability of 0.5 was added to the fully connected layer. Finally, low-risk probabilities were calculated using the softmax function. Using the same CNN architecture, 11 models were separately trained with each of the 11 selected slices and then used to extract deep features from each image. The performances of 11 models were validated using five-fold cross-validation (Supplementary Table S1). We could not verify them with the external validation cohort since there was no 6MWD information, but performance can be inferred by whether our survival prediction model works well. Nevertheless, the fact that our CNN models for extracting deep features have not been tested well enough to prove their performances is a limitation of our model. Deep features were obtained by normalizing the information of the last fully connected layer; we designed and compared models with various sizes of fully connected layers of 128, 256, 512, and 1024. The CNN designed for extracting deep features was implemented in Keras with a Theano backend.

**Table 1:** Baseline clinical characteristics and mortality data.

	Discovery cohort				External validation cohort	*p-*value (discovery vs external validation cohort)
	6MWD > 440 m	6MWD ≤ 440 m	*p-*value (> 440 m vs. ≤ 440 m)	Total		
Patients (N)	157	187		344	102	
Mortality* (N)	12 (7.6%)	31 (16.6%)	0.018	43 (12.5%)	18 (17.6%)	0.185
Follow-up (months)	84.8 (2, 149)	57.1 (2, 148)	< 0.001	69.8 (2, 149)	32.9 (2, 47)	< 0.001
Age (years)	64.0 ± 7.0	69.8 ± 7.4	< 0.001	67.2 ± 7.8	68.3 ± 8.0	0.601
**Gender**			0.151			0.006
Male (N)	156 (99.4%)	182 (97.3%)		338 (98.3%)	95 (93.1%)	
Female (N)	1 (0.6%)	5 (2.7%)		6 (1.7%)	7 (6.9%)	
Height	167.0 ± 5.4	164.4 ± 6.3	< 0.001	165.6 ± 6.1	–	
Weight	66.0 ± 8.7	61.2 ± 10.1	< 0.001	63.4 ± 9.8	–	
6MWD (meters)**	499.7 (441, 652)	368.8 (120, 440)	< 0.001	428.6 (120, 652)	–	
**PFT**						
FEV_1_ (% predicted)	57.9 ± 16.5	53.5 ± 18.0	0.022	55.5 ± 17.4	47.9 ± 21.3	< 0.001
FVC (% predicted)	87.5 ± 17.4	84.6 ± 17.8	0.137	85.9 ± 17.7	65.1 ± 22.9	< 0.001
FEV_1_/FVC	47.7 ± 9.8	43.9 ± 11.3	0.001	45.7 ± 10.8	53.0 ± 11.1	< 0.001
**Gold stage**			< 0.001			< 0.001
Stage I (N)	23 (14.7%)	13 (7.0%)		36 (10.5%)	5 (4.9%)	
Stage II (N)	84 (53.5%)	87 (46.5%)		171 (49.7%)	37 (36.3%)	
Stage III (N)	44 (28.0%)	70 (37.4%)		114 (33.1%)	42 (41.2%)	
Stage IV (N)	6 (3.8%)	17 (9.1%)		23 (6.7%)	18 (17.6%)	

Continuous variables are presented as mean ± standard deviation and categorical data are presented as the number of patients with percentages in parentheses. ‘Follow-up’ and ‘6MWD’ are presented as the mean with minimum and maximum values in parentheses.

*6MWD* six-minute walk distance, *PFT* pulmonary function test, *FEV*_*1*_ forced expiratory volume in 1 s, *FVC* forced vital capacity, *GOLD* Global Initiative for Chronic Obstructive Lung Disease.

*Overall survival was investigated as study end-point, which is defined as the time until death from any cause.

**6MWD information was only in the discovery cohort, and Supplementary Figure S2 shows the distribution of it.

### Survival analysis and statistical comparison

To predict overall survival in COPD patients, random survival forest (RSF) with deep features of each slice was performed. Our deep features were predominantly black box features so that it was difficult to effectively reduce multiple collinear and correlated predictors that could produce unstable estimates and might overfit predictions. RSF is a censored data extension of the Random Forest method, where the ensemble survival function is constructed by aggregating tree-based estimator^31^. We expected that RSF would effectively ensemble the features extracted from our censored data to predict mortality. RSF analysis was implemented using the randomForestSRC R package with default settings. RSF calculated a survival curve for each patient, grew a forest using log-rank splitting, and then averaged the results of the forest, obtaining a stable result. Using RSF, we could predict the survival and cumulative hazard function of individuals. The performance of the proposed deep radiomics-based survival prediction model was evaluated in two independent datasets: (1) the discovery cohort (KOLD^26^) and (2) the external validation cohort (Malaysia^28^). For a quantified comparison, we computed the concordance probability (C-index) and time-dependent area under the receiver operating characteristic (ROC) curve (AUC). C-index is the frequency of concordant pairs among all pairs of subjects and can be used to measure and compare the discriminative power of a risk prediction model. The time-dependent AUC has incorporated time dependency in AUC in time-event data for individuals instead of using the standard ROC curve approach^32,33^, dealing with censored data and yielding different values of AUC at each time point. Internal validation used ROC curves for 3- and 5-year survival, but external validation was not calculated time-dependent AUC because of its follow-up duration. The time-dependent AUC was implemented using the timeROC R package.

## Results

There were two groups of patients: (1) a discovery cohort with 344 patients from KOLD cohort^26^ and (2) an external validation cohort with 102 patients from Penang General Hospital in Malaysia^28^. Baseline clinical characteristics and mortality data are summarized in Table 1. Overall survival was investigated as the study end-point, which is defined as the time until death from any cause, and Supplementary Figure S1 shows the Kaplan–Meier survival curves of two cohorts.

### Internal validation

Deep feature-based survival analysis was performed via five-fold cross-validation of the discovery cohort to determine which features provide more high-level medical image information and predictive patterns. In order to find the optimal number of features suitable for predicting overall survival in COPD patients, we trained the binary classifier of 6MWD test results with various sizes of fully connected layers of 128, 256, 512, 1024, and then extracted deep features using the representative 11 CT slices. Mortality prediction performance was evaluated using combinations of deep features of each slice, which were obtained by pooling (e.g., for the combination of C1 and C2 with 256 deep features, 512 deep features were used as the input to the RSF). We performed RSF for all combinations of 11 slices. Depending on the number of selected samples, the following numbers of combinations were produced, and RSF was performed for a total of 2047 combinations: 1- and 10-selection, 11 combinations; 2- and 9-selection, 55 combinations; 3- and 8-selection, 165 combinations; 4- and 7-selection, 330 combinations; 5- and 6-selection, 462 combinations; 11-selection, 1 combination. The number of *k*-element (*k*-selection) combinations of *n* objects without repetition is ${}_{n}^{ } C_{k} = n!/k!(n - k)!$—for example, 2- and 9-selection lead to ${}_{11}^{ } C_{2} = {}_{11}^{ } C_{9} = 11!/2!9! = 55$ distinct combinations. Eventually, the experiments were performed with size of deep features of 128, 256, 512, and 1024, and the performance of all combinations of 11 representative CT slices in each size of deep features was compared and top 5 combinations were summarized in Table 2. The performance of 256 deep features was superior, and the highest C-index of 0.8008 (95% CI, 0.7642–0.8373) was obtained by combining the coronal slice of the vertebral body and two sagittal slices of the left/right lung (C1 + S1 + S2). At this combination, AUC for 3- and 5-year survival was and 0.8878 (95% CI, 0.7900–0.9856) and 0.8411 (95% CI, 0.7901–0.8922), respectively.

**Table 2:** Survival prediction performance of deep features in the discovery and external validation cohort.

No. of features	Combinations	Internal validation			External validation
		C-index (95% CI)	AUC for 3-year survival	AUC for 5-year survival	C-index (95% CI)
128	1. C3 + S2	0.7753 (0.7411, 0.8095)	0.8042 (0.6254, 0.9831)	0.8364 (0.7895, 0.8833)	0.6502 (0.6394, 0.6610)
	2. S2	0.7745 (0.7237, 0.8253)	0.8198 (0.6552, 0.9844)	0.8197 (0.7639, 0.8756)	0.6577 (0.6466, 0.6688)
	3. C1 + C3 + S2 + A6	0.7711 (0.7210, 0.8212)	0.7922 (0.5860, 0.9984)	0.8247 (0.7889, 0.8605)	0.6424 (0.6268, 0.6580)
	4. C1 + C3 + S2	0.7706 (0.7291, 0.8121)	0.7928 (0.5943, 0.9913)	0.8311 (0.7850, 0.8772)	0.6217 (0.6085, 0.6348)
	5. C2 + C3 + S2	0.7676 (0.7209, 0.8143)	0.8175 (0.6404, 0.9945)	0.8334 (0.7888, 0.8779)	0.5803 (0.5656, 0.5951)
**256**	**1. C1 + S1 + S2**	**0.8008 (0.7642, 0.8373)**	**0.8878 (0.7900, 0.9856)**	**0.8411 (0.7901, 0.8922)**	**0.7156 (0.7024, 0.7288)**
	2. C1 + C2 + S1 + S2 + A4	0.7959 (0.7682, 0.8236)	0.8642 (0.7638, 0.9646)	0.8400 (0.7865, 0.8935)	0.7130 (0.7009, 0.7252)
	3. S1 + S2 + A4 + A5	0.7948 (0.7655, 0.8240)	0.8543 (0.7638, 0.9448)	0.8390 (0.7871, 0.8954)	0.6742 (0.6625, 0.6858)
	4. S1 + S2 + A1 + A4 + A6	0.7938 (0.7719, 0.8157)	0.8337 (0.7101, 0.9573)	0.8371 (0.7819, 0.8923)	0.6404 (0.6274, 0.6533)
	5. S1 + S2 + A4	0.7930 (0.7593, 0.8267)	0.8338 (0.7259, 0.9416)	0.8512 (0.8007, 0.9017)	0.6400 (0.6274, 0.6527)
512	1. C1 + C3 + S2 + A4	0.7750 (0.7432, 0.8068)	0.8310 (0.7301, 0.9319)	0.8254 (0.7892, 0.8616)	0.6713 (0.6600, 0.6825)
	2. C1 + C3 + S1 + S2 + A4	0.7716 (0.7346, 0.8085)	0.8356 (0.7027, 0.9685)	0.8251 (0.7684, 0.8817)	0.6714 (0.6606, 0.6822)
	3. C1 + C3 + S1 + S2 + A1 + A3	0.7659 (0.7343, 0.7975)	0.8194 (0.7260, 0.9129)	0.8078 (0.7926, 0.8230)	0.6277 (0.6153, 0.6401)
	4. C1 + C3 + S1 + S2 + A1 + A2 + A3 + A6	0.7657 (0.7430, 0.7884)	0.8261 (0.7294, 0.9228)	0.7891 (0.7606, 0.8177)	0.6660 (0.6533, 0.6786)
	5. C1 + C3 + S2	0.7657 (0.7250, 0.8064)	0.8338 (0.7259, 0.9416)	0.8238 (0.7861, 0.8615)	0.6411 (0.6273, 0.6549)
1024	1. S2 + A2 + A5	0.7813 (0.7333, 0.8294)	0.7255 (0.5634, 0.8877)	0.8257 (0.7969, 0.8544)	0.6805 (0.6186, 0.7424)
	2. S2 + A2 + A5 + A6	0.7765 (0.7439, 0.8090)	0.7712 (0.6367, 0.9056)	0.8094 (0.7517, 0.8672)	0.6515 (0.6024, 0.7007)
	3. A2 + A5	0.7744 (0.7310, 0.8179)	0.7990 (0.7072, 0.8908)	0.8181 (0.7318, 0.9045)	0.6305 (0.5686, 0.6924)
	4. S2 + A2	0.7729 (0.7181, 0.8278)	0.7864 (0.6722, 0.9006)	0.8300 (0.7413, 0.9187)	0.6119 (0.5226, 0.7013)
	5. C1 + C3 + S2 + A5	0.7729 (0.7337, 0.8120)	0.6881 (0.4501, 0.9261)	0.8167 (0.7563, 0.8770)	0.6844 (0.6664, 0.7023)

*C1, C2, C3* coronal slices at the vertebral body, center of the tracheal carina, and superior vena cava, respectively; *S1, S2* sagittal slices at the right and left lung, respectively; *A3* axial slice at the center of the tracheal carina; *A1, A2, A4, A5, A6* upper two and lower three axial slices at 2-cm intervals at the center of the tracheal carina, respectively. Bold values denote best-performed combinations of each column.

### External validation

The deep radiomics model was evaluated against an external validation cohort (102 CT scans). Top 5 combinations of deep features were used to evaluate the proposed method since they had the best results on internal validation (Table 2). In the external validation, the Rank 1 combination of the internal validation showed also the best C-index of 0.7156 (95% CI, 0.7024–0.7288).

## Discussion

The current study found that a deep radiomics approach for survival prediction in COPD patients was feasible and showed acceptable performance, which was confirmed by concordant results in an external validation cohort. The deep features from the CNN model using COPD patients’ chest CT data were found to be significant and independent predictors of mortality in both the discovery and external validation groups.

Many methodologies using quantitative CT features for quantitative assessment of different COPD components have been studied, but few studies have adapted an integrative approach^3,34,35^. A deep radiomics approach using a CNN to extract many learned features from chest CT images may be helpful in developing clinically useful decision support models.

In this study, a chest CT-based deep radiomics approach with a CNN was used for the first time to predict survival in COPD patients. The CNN performed binary classification based on 6MWD results (> 440 m or not). In the discovery and external validation cohorts, with combining the coronal slice of the vertebral body and two sagittal slices of the left/right lung (C1 + S1 + S2, 256 × 3 deep features), deep radiomics for survival prediction achieved C-indices of 0.8008 (95% CI, 0.7642–0.8373) and 0.7156 (95% CI, 0.7024–0.7288), respectively, and AUC for 3- and 5-year survival was and 0.8878 (95% CI, 0.7900–0.9856) and 0.8411 (95% CI, 0.7901–0.8922), respectively. Comparing performances of the best combination to using all slices for each number of deep features (Supplementary Table S2), it is meaningful to use slices selectively. Among studies using quantitative CT features as a predictor of survival in COPD patients^36–38^, Cho et al.^38^ reported the performance using the same datasets of ours. Five features were selected as the final radiomics signature; (1) a percentage of low attenuation area; (2) airway wall thickness of 6th generation bronchus at an internal perimeter of 10 mm; (3) heterogeneity of percentage wall area; (4) heterogeneity of airway wall thickness at an internal perimeter of 10 mm; (5) average pulmonary vessel cross-sectional area measured at 18 mm from the pleural surface. C-indices of five final radiomics signature were 0.699, 0.531, 0.615, 0.542, and 0.605, respectively, and the combinations of radiomics signature were 0.774. In the same datasets, our deep feature-based survival prediction model outperformed compared to the quantitative CT features. Moll et al.^37^ proposed a survival prediction model using a combination of clinical and quantitative CT features which reported a C-index ≥ 0.7 and showed 6MWD as the most important predictor. In our discovery cohort, a prediction model with only 6MWD achieved C-index of 0.6072 (95% CI, 0.6014–0.6130), and our model surpassed it. The mortality prediction performance of our model was externally validated in a separate group of patients. Patient enrollment for the discovery cohort began much earlier (June 2005) than for the external validation cohort (September 2013), which inevitably led to differences in follow-up duration (mean, 69.8 vs. 32.9 months). Although there were some differences in characteristics between two groups including a considerable difference in follow-up duration, external validation was performed to demonstrate the generalizability and transportability of our model.

Both spirometry and multidimensional indices are limited in that they cannot fully represent the type and range of morphologic alterations that may be detectable before functional parameters begin to deteriorate. With a deep radiomics approach, essential information related to phenotypic heterogeneity and pathophysiology may be learned from medical images and used to improve medical decision-making in COPD patients. In the current study, the deep radiomics approach was confined to survival prediction in COPD patients. However, we believe that a deep radiomics approach could potentially be applied to other facets of COPD, such as reliable phenotyping, predicting acute exacerbation, and monitoring treatment response.

The current study is subject to several limitations. First, although our deep feature-based survival prediction model has been integrally analyzed in the discovery and external validation cohorts, a larger study population would have been beneficial, especially in the external validation group. Second, because all patients included in this study were Asian, the results may not be applicable to patients of other ethnicities. However, a major strength of the current research is that the positive discovery group findings were externally validated using a group of patients of a different nationality. That said, further validation of the findings in a larger-scale study with patients of different ethnicities is warranted. Third, the proportion of males in our discovery and external validation cohorts amount to 98.3% and 93.1% respectively, resulting in the striking gender imbalance. The imbalance can partly be explained by the cultural environment where the smoking rate in men (36.7% in South Korea, and 43.0% in Malaysia) is overwhelmingly higher than that of women (7.5% in South Korea, and 1.4% in Malaysia) in general population^39,40^, and sexual difference in COPD prevalence^40–42^. Nevertheless, we should admit that our model is likely to be bias/specific for male lungs due to the anatomical and physiological differences between males and females. Fourth, our model requires that 11 representative CT slices should be manually selected by an expert. It can be an additional workload for the radiologists, and it has the possibility of incorrect selection of them. The development of a deep learning-based system for detecting anatomic landmarks could be helpful. Fifth, the deep radiomics features were predominantly black box features; therefore, they need to be interpreted by case review and other technical methods. Lastly, the survival prediction performance of the deep radiomics model was not directly compared with that of other various clinical risk-scoring systems such as the Body mass index, airflow Obstruction, Dyspnea, and Exercise (BODE) index, and the incremental value of the deep radiomics model was not fully investigated. In the future, evaluating the relationship between deep features and traditional lung density measurements would be useful and potential added value.

In conclusion, a deep radiomics approach for survival prediction was feasible. The performances of 20 models (top 5 combinations in each size 128, 256, 512, and 1024 of deep features) were compared, and the highest C-index of 0.8088 8008 (95% CI, 0.7642–0.8373) was obtained by combining 256 features each from a coronal slice and two sagittal slices (C1 + S1 + S2), as confirmed by concordant results (C-index, 0.7156; 95% CI, 0.7024–0.7288) in an external validation group. The models with 256 deep features performed superior, C1 + S1 + S2 performed best, but there is a risk of false discovery because the differences in the results of different combinations are insignificant.

## Supplementary Information

Supplementary Information.

## Data Availability

The datasets generated during and/or analysed during the current study are available from the corresponding author on reasonable request.
