# Supplementary material for: Deep radiomics-based survival prediction in patients with chronic obstructive pulmonary disease
Source: Sci Rep. 2021 Jul 26;11:15144. doi: 10.1038/s41598-021-94535-4 (PMC8313653; doi:10.1038/s41598-021-94535-4)
Supplement: Supplementary file 1 — Supplementary Information. [file 41598_2021_94535_MOESM1_ESM.docx]

Deep radiomics-based survival prediction in patients with chronic obstructive pulmonary disease

**Jihye Yun**^1^, PhD; Young Hoon Cho^2^, MD; Sang Min Lee^1^, MD; Jeongeun Hwang^3^, PhD; Jae Seung Lee^4^, MD; Yeon-Mok Oh^4^, MD; Sang Do Lee^4^, MD; Li-Cher Loh^5^, MD; Choo-Khoon Ong^5^, MD; **Joon Beom Seo**^1,*^, MD; **Namkug Kim**^1,6,*^, PhD

^1^Department of Radiology, Asan Medical Center, University of Ulsan College of Medicine

^2^Department of Radiology, Korea University Guro Hospital, Korea University College of Medicine

^3^Department of Medicine, University of Ulsan College of Medicine

^4^Department of Pulmonary and Critical Care Medicine and Clinical Research Center for Chronic Obstructive Airway Diseases, Asan Medical Center, University of Ulsan College of Medicine

^5^Department of Medicine, RCSI & UCD Malaysia Campus

^6^Department of Convergence Medicine, Asan Medical Institute of Convergence Science and Technology, Asan Medical Center, University of Ulsan College of Medicine

^*^ These two authors contributed equally as the corresponding authors.

^+^These two authors contributed equally to this work.

Supplementary Appendix I

We validated the method using multiple datasets from the discovery and external validation cohorts. The two cohorts had different follow-up times, and there were significant differences in their estimated cumulative survivals.


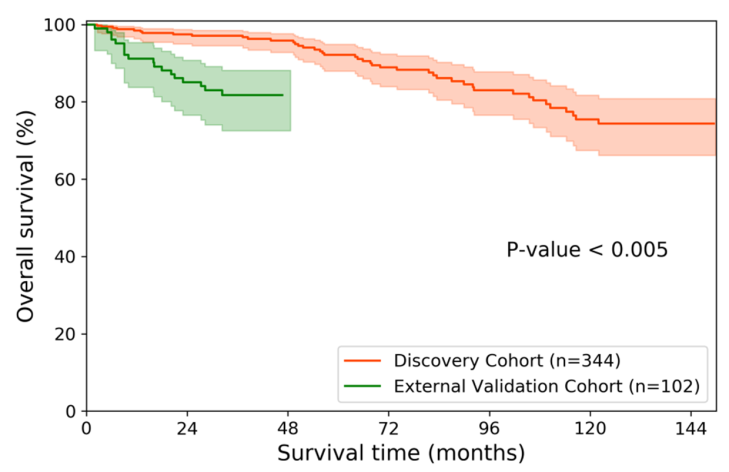


Figure S1. Kaplan-Meier survival curves for discovery and external validation cohorts.

Supplementary Appendix II

We designed and trained a CNN-based binary classifier to obtain high-level representative information from chest computed tomography (CT) images that can predict mortality in patients with chronic obstructive pulmonary disease, and then high-throughput image features (i.e., deep radiomics) were extracted from the last fully connected layer. Our CNN-based binary classifiers were trained based on a six-minute walk distance (6MWD) testing result (> 440m or not) which is one of several factors associated with low one-year mortality.^30^ The distribution of 6MWD in our discovery cohort is as follows:


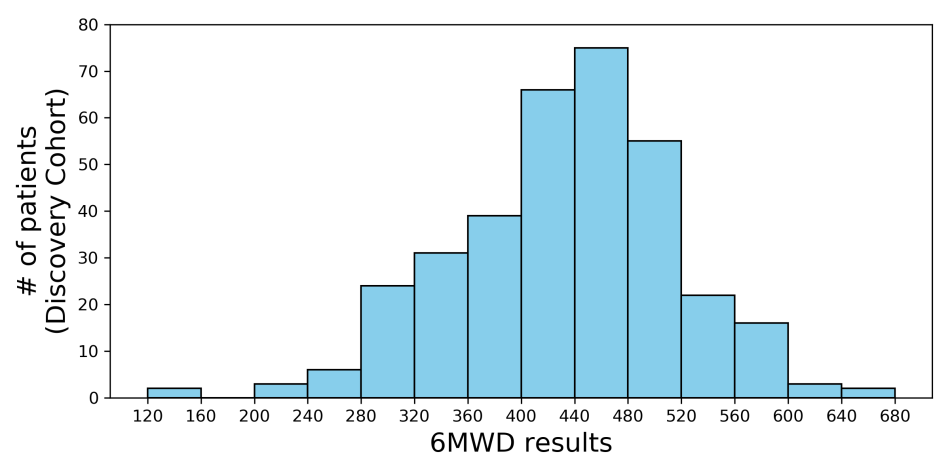


Figure S2. Distribution of 6MWD in the discovery cohort.

With the value of 440m, it consisted of 157 patients with 6MWD over 440m and 187 patients with 6MWD less than 440m, and the Kaplan-Meier-estimated cumulative survivals of these two groups compared in Figure 1(B).

Supplementary Appendix III

Using the same CNN architecture (Figure 4), 11 models were separately trained with each of the 11 selected slices, and then they were used to extract deep features from each image. We validated these 11 models using five-fold cross-validation.

Table S1. Performances of CNN-based Binary Classifier for Extraction of Deep Features

| Input slice of  6MWD classifier | Accuracy (95% CI) | | | |
| --- | --- | --- | --- | --- |
|  | # of deep features  = 128 | # of deep features  = 256 | # of deep features  = 512 | # of deep features  = 1024 |
| C1 | 65.40 (61.47, 69.33) | 63.67 (59.59, 67.74) | 64.25 (60.02, 68.49) | 63.07 (58.04, 68.11) |
| C2 | 62.78 (59.70, 65.86) | 62.22 (60.09, 64.35) | 65.11 (59.24, 70.98) | 63.96 (57.96, 69.96) |
| C3 | 67.44 (63.12, 71.75) | 65.71 (61.87, 69.55) | 64.53 (60.29, 68.77) | 64.23 (61.49, 66.97) |
| S1 | 63.36 (60.18, 66.53) | 60.17 (58.47, 61.88) | 62.79 (61.10, 64.48) | 63.37 (60.73, 64.28) |
| S2 | 60.76 (59.22, 62.30) | 60.76 (56.38, 65.14) | 59.30 (54.03, 64.57) | 61.62 (58.97, 66.00) |
| A1 | 65.68 (62.62, 68.73) | 63.35 (59.94, 66.77) | 66.86 (63.50, 70.22) | 64.80 (60.75, 68.86) |
| A2 | 61.61 (56.03, 67.19) | 62.19 (58.47, 65.90) | 58.72 (55.51, 61.92) | 62.77 (56.31, 69.23) |
| A3 | 63.64 (58.96, 68.31) | 62.77 (59.00, 66.55) | 63.65 (57.13, 70.17) | 61.91 (58.00, 65.81) |
| A4 | 63.65 (58.84, 68.46) | 61.34 (58.51, 64.17) | 65.69 (63.47, 67.90) | 59.29 (55.89, 62.70) |
| A5 | 64.51 (58.46, 70.55) | 63.66 (59.37, 67.94) | 65.69 (63.79, 67.58) | 66.26 (62.20, 70.31) |
| A6 | 61.33 (58.14, 64.51) | 61.90 (58.77, 65.03) | 62.50 (58.44, 66.56) | 62.50 (60.15, 64.85) |
|  | 63.65 (62.45, 64.85) | 6252 (61.60, 63.44) | 6355 (62.02, 65.08) | 63.07 (61.99, 64.15) |

Supplementary Appendix IV

Comparing performances of the best combination to using all slices for each number of deep features, it is meaningful to use slices selectively.

Table S2. Performance Comparison between Best combination and Using All Slices

| # of features | Performance of top 1 combination | | Performance of all 11 slices | |
| --- | --- | --- | --- | --- |
|  | Top 1 combination | C-index (95% CI) | C-index (95% CI) | Rank |
| 128 | C3+S2 | 0.7753 (0.7411, 0.8095) | 0.7084 (0.6510, 0.7658) | 1,576 |
| 256 | C1+S1+S2 | 0.8008 (0.7642, 0.8373) | 0.7704 (0.7515, 0.7894) | 376 |
| 512 | C1+C3+S2+A4 | 0.7750 (0.7432, 0.8068) | 0.7390 (0.7043, 0.7737) | 530 |
| 1024 | S2+A2+A5 | 0.7813 (0.7333, 0.8294) | 0.7224 (0.6569, 0.7879) | 1,251 |
